# Assessment of antigenic difference of equine influenza virus strains by challenge study in horses

**DOI:** 10.1111/irv.12418

**Published:** 2016-08-09

**Authors:** Takashi Yamanaka, Manabu Nemoto, Hiroshi Bannai, Koji Tsujimura, Takashi Kondo, Tomio Matsumura, Sarah Gildea, Ann Cullinane

**Affiliations:** ^1^ Equine Research InstituteJapan Racing AssociationShimotsukeTochigiJapan; ^2^ Virology UnitIrish Equine CentreJohnstownNaasCo. KildareIreland

**Keywords:** challenge study, equine influenza, H3N8, inactivated whole vaccine

## Abstract

We previously reported that horse antiserum against the Japanese equine influenza vaccine virus, A/equine/La Plata/1993 (LP93) exhibited reduced cross‐neutralization against some Florida sublineage Clade (Fc) 2 viruses, for example, A/equine/Carlow/2011 (CL11). As a result, Japanese vaccine manufacturers will replace LP93 with A/equine/Yokohama/aq13/2010 (Y10, Fc2). To assess the benefit of updating the vaccine, five horses vaccinated with inactivated Y10 vaccine and five vaccinated with inactivated LP93 were challenged by exposure to a nebulized aerosol of CL11. The durations of pyrexia (≥38.5°C) and other adverse clinical symptoms experienced by the Y10 group were significantly shorter than those of the LP93 group.

## 1 Introduction

Equine influenza (EI) caused by the H3N8 subtype of equine influenza A virus (EIV), characterized by coughing, nasal discharge, and pyrexia is considered the most important infectious respiratory disease of horses.^1^ Many outbreaks occur in vaccinated horse populations when the vaccine viruses are not closely antigenically related to those circulating in the field.^1^ Therefore, it is necessary to periodically review the composition of vaccines and evaluate their efficacy with epidemiologically relevant viruses. EIV diverged genetically into the Eurasian and American lineages in the 1980s, and the American lineage subsequently diverged into the Kentucky, Argentine, and Florida sublineages, with the Florida sublineage dominating in recent years. Most recently, the Florida sublineage diverged into two clades, Florida sublineage clade (Fc) 1 and Fc2. The clades are antigenically distinguishable and since 2010 the World Organisation for Animal Health (OIE) has recommended that EI vaccines contain viruses representative of both clades.^1^ However, as of April 2016, Japanese EI vaccines did not contain Fc2 virus. We previously reported that horse antiserum raised against the Japanese vaccine virus, A/equine/La Plata/1993 (LP93, Argentine sublineage), showed limited cross‐neutralization against some Fc2 viruses, for example, A/equine/Carlow/2011 (CL11) carrying the substitution (A144V) in antigenic site A of the hemagglutinin (HA).^2^ Reportedly, the majority of recent isolates in some European countries carries the substitution.^3^ Therefore, Japanese vaccine manufacturers will replace LP93 with an Fc2 strain (A/equine/Yokohama/aq13/2010: Y10), which shows the satisfactory characters for manufacturing vaccines (propagation ability, immunogenicity in mice, etc.).^4^ Here, we compared the level of protection afforded by vaccines containing either inactivated Y10 or LP93, in horses experimentally challenged with CL11.

## 2 Materials and Methods

### 2.1 Viruses, vaccinations, and animals

The EIVs (LP93, Y10, and CL11) were prepared as previously described.^2^, ^5^ Monovalent inactivated (0.05% formaldehyde) vaccines were prepared using 400 chicken RBC hemagglutinating units/dose. Ten 1‐year‐old influenza‐naïve Thoroughbred horses were randomly divided into two groups of five, each group receiving either Y10 or LP93 vaccine. Horses were vaccinated twice, one month apart, by intramuscular injection of monovalent non‐adjuvanted vaccine.

### 2.2 Challenge study

Two horses in each group (horses 1 and 2 of Y10 group, horses 6 and 7 of LP93 group) were experimentally challenged with 10^9.4^ 50% egg infectious dose (EID_50_) of CL11 per horse, 2 weeks after the second vaccination as previously described.^5^ The remaining three horses in each group (horses 3, 4, and 5 of Y10 group, horses 8, 9, and 10 of LP93 group) were similarly challenged 4 weeks after second vaccination.

Rectal temperatures were measured daily for 14 days post‐challenge, and pyrexia was defined as ≥38.5°C.^6^ Nasopharyngeal swabs were collected daily for 14 days after the challenge and virus isolation conducted as previously described.^5^ Virus shedding was defined as ≥10^0.7^EID_50_/200 μL. Sera were collected on the day of the primary vaccination and on the challenge day. The experimental protocols were approved by the Animal Care Committee of Equine Research Institute of Japan Racing Association.

### 2.3 Serological tests

Sera were treated with trypsin‐heat‐potassium metaperiodate to remove non‐specific inhibitors.^7^ Hemagglutination inhibition (HI) and virus neutralization (VN) titers were determined as previously reported.^2^, ^7^

### 2.4 Data analysis

The mean rectal temperatures were analyzed with a two‐way analysis of variance and post hoc Fisher LSD test between the groups on each day. The mean durations (days) of pyrexia and virus shedding between the groups were compared using an unpaired Student's *t*‐test. All statistical analyses were performed with graphpad prism 6 for Windows (GraphPad Software, Inc, San Diego, CA, USA). A level of *P*<.05 was considered significant. When geometric mean (GM) HI and VN titers were calculated, titers at <8 were provisionally considered four in this study.

## 3 Results

Horses were seronegative by HI and VN on the day of primary vaccination. The HI and VN titers on the challenge day (Day 0) are represented in Table 1. While the GM HI titers against CL11 of sera collected from Y10 and LP93 groups were similar (27.9 and 24.3, respectively), the GM VN titers against CL11 of sera collected from the Y10 group (48.5) were approximately 4.6‐fold higher than the LP93 group (10.6). Horse 5 (Y10 group) showed no antibody response after vaccination even against the homologous virus, suggesting that the horse was a poor vaccine responder. By excluding Horse 5, the GM VN titer against CL11 was 8.5 times higher for Y10 group compared with the LP93 group (Table 1).

**Table 1:** Hemagglutination inhibition and virus neutralization titers against vaccine viruses (Y10 and LP93) and the challenge virus (CL11) on the day of challenge, Day 0

Group	Horse	HI titer			VN titer		
		Antigen			Virus		
		Y10	LP93	CL11	Y10	LP93	CL11
Y10	Horse 1	64	64	128	256	256	128
	Horse 2	32	32	64	64	64	64
	Horse 3	32	16	16	128	64	64
	Horse 4	64	32	32	256	128	128
	Horse 5	<8	<8	<8	<8	<8	<8
	GMT^a^^,^ b	27.9 (45.3)	21.1 (32.0)	27.9 (45.3)	73.5 (152.2)	55.7 (107.6)	48.5 (90.5)
LP93	Horse 6	64	64	64	128	256	16
	Horse 7	32	32	32	64	128	8
	Horse 8	64	64	32	256	256	32
	Horse 9	32	32	16	128	128	8
	Horse 10	16	16	8	64	128	<8
	GMT	36.8	36.8	24.3	111.4	168.9	10.6

a Geometric mean titer.

b The GMTs of the Y10 group excluding Horse 5 are represented in parentheses. Horse 5 was considered a poor vaccine responder (see the text).

**Figure 1:**
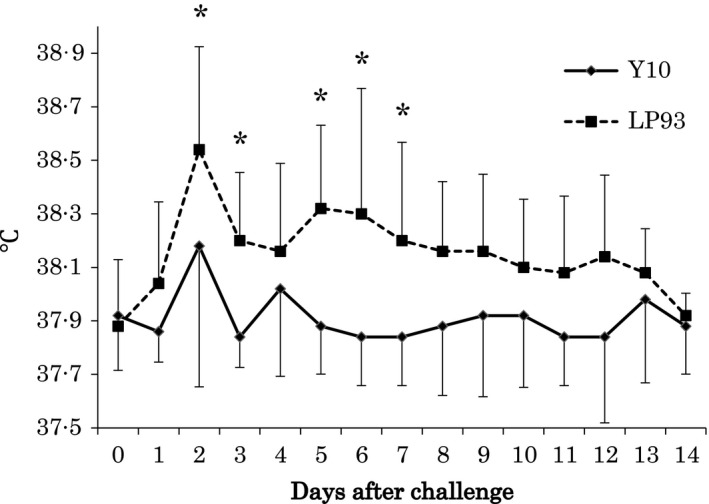
Daily mean (±SD) rectal temperatures of horses in the Y10 and LP 93 groups (*P<.05)

The mean rectal temperatures of the Y10 group were significantly lower than LP93 group for 5 days (Fig. 1), and the mean duration of pyrexia of the Y10 group (days±SD) (0.4±0.9) was significantly shorter than the LP93 group (3.0±1.9, *P*=.023). Whereas all the horses in LP93 group exhibited virus shedding after challenge, there was no evidence of virus shedding by two horses in the Y10 group (Table 2). However there was no significant difference in the mean durations (days±SD) of virus shedding between the two groups (*P*=.294).

**Table 2:** Virus isolation and quantification (log_10_EID_50_/200 μL) by egg culture of nasopharyngeal swabs collected from the experimentally infected horses

Days after challenge	Y10 group					LP93 group				
	Horse 1	Horse 2	Horse 3	Horse 4	Horse 5	Horse 6	Horse 7	Horse 8	Horse 9	Horse 10
0	–a	–	–	–	–	–	–	–	–	–
1	–	–	–	1.5	1.5	≤0.7	–	2.5	1.5	1.7
2	–	–	–	≤1.0	≤1.3	–	3.5	–	1.7	1.7
3	–	–	–	–	2.5	–	≤0.7	–	–	≤0.7
4	–	–	–	–	1.5	–	≤0.7	–	≤1.0	≤0.7
5	–	–	–	–	≤1.3	–	2.0	–	–	–
6	2.7	–	–	–	–	≤0.7	–	–	–	–
7	≤0.7	–	–	–	–	≤1.0	–	–	–	–
8	–	–	–	–	–	–	–	–	–	–
9	–	–	–	–	–	–	–	–	–	–
10	–	–	–	–	–	–	–	–	–	–
11	–	–	–	–	–	–	–	–	–	–
12	–	–	–	–	–	–	–	–	–	–
13	–	–	–	–	–	–	–	–	–	–
14	–	–	–	–	–	–	–	–	–	–

a <0.7 (No EIV was isolated from a nasopharyngeal swab).

## 4 Discussion

We previously reported that the VN titers of horse LP93 antiserum were ≥eightfold lower against Fc2 viruses with HA A144V substitution than against homologous virus, or Fc2 viruses lacking A144V substitution.^2^ In contrast, these differences narrowed to fourfold when the antibody titers were measured by HI test (Table S1). A virus is traditionally considered “vaccine virus‐like” if the antiserum raised against the vaccine virus exhibits less than an eightfold reduction in HI titer against the virus compared with the titer of homologous vaccine virus.^8^, ^9^ According to these criteria, the HI test results indicated that the antigenic difference between LP93 and Fc2 viruses with A144V substitution was not significant. Therefore, it was essential to investigate whether or not the eightfold antigenic difference by VN tests had an impact on vaccine efficacy in horses.

On Day 0, that is, after two vaccinations, the GM HI titers of both study groups against CL11 were similar, but there was a fourfold difference in HI titers 14 days after the challenge infection. In ferrets, administration of a booster dose of influenza virus causes a broadening of the immune response.^8^, ^10^ Consistent with this, the administration of two doses of LP93 vaccine to horses broadened the immune response elicited to CL11 as measured by HI assay. However, the approximately 4.6‐fold difference in GM VN titers against CL11 between the groups was maintained. This suggests that VN antibodies raised against the vaccine virus (LP93) are more likely to sustain homologous virus specificities than HI antibodies, even after repeated vaccinations.

On exclusion of the poor responder to vaccination (Horse 5), the homologous GM HI and VN titers were similar between groups indicating that Y10 and LP93 vaccines are of similar immunogenicity. Poor vaccine responders have been identified in the field previously and they are believed to have the potential to shed large amounts of virus.^11^, ^12^ Indeed, the period of virus shedding of Horse 5 was the longest among all the horses, and this horse was the only member of Y10 group to exhibit pyrexia (Table S2). Despite the poor vaccine responder in Y10 group, the mean duration and degree of pyrexia were less than that exhibited by the LP93 group. These findings correlated with the higher GM VN titer of Y10 group against the challenge virus compared with the LP93 group. The duration of virus shedding was not statistically different between the groups. However, on exclusion of Horse 5, the poor responder, the duration of virus shedding in the Y10 group was significantly shorter than in the LP93 group (*P*=.041). While all the horses of the LP93 group showed some clinical signs after challenge, the mean duration of clinical signs in the Y10 group was significantly shorter than in the LP93 group (Table S3, *P*=.045), indicating the superior efficacy of the Y10 vaccine compared with the LP93 vaccine.

In summary, this study demonstrates the superior efficacy of the Y10 vaccine compared with the LP93 vaccine, against an Fc2 virus carrying HA A144V substitution. The increase in protection against virus challenge may be due to the higher VN titers against CL11 induced by vaccination with the Y10 vaccine. As similar HI antibody levels were induced by both vaccines, it appears that VN antibody measurement is more likely, than HI, to identify antigenic differences in influenza viruses, which could affect vaccine efficacy in horses.

## Competing Interests

The authors declare that they have no competing interests.
