# Supplementary material for: Assessment of antigenic difference of equine influenza virus strains by challenge study in horses
Source: Influenza Other Respir Viruses. 2016 Aug 9;10(6):536–9. doi: 10.1111/irv.12418 (PMC5059955; doi:10.1111/irv.12418)
Supplement: Supplementary file 1 [file IRV-10-536-s001.docx]

| Table S1. Cross reactivity in HI assays of horse antisera collected 14 days after experimental infection with LP93 or Y10. These antisera were used in VN assays in our previous study.^2^ | | | | | | | | | | | | | | | | |
| --- | --- | --- | --- | --- | --- | --- | --- | --- | --- | --- | --- | --- | --- | --- | --- | --- |
| Antigen | Horse antiserum raised to | |  | HA1 amino acid substitutions | | | | | | | | | | | | |
|  | LP93 | Y10 |  | 5 | 7 | 30 | 48 | 58 | 103 | 112 | 144 | 169 | 189 | 272 | 282 | 312 |
| LP93 | **512 (512)** | 128 (51) |  | T | G | T | I | V | P | V | A | P | N | A | P | K |
| A/equine/Richmond/1/2007^a^ | 512 (512) | 256 (256) |  | I | N | S | M | I | P | V | A | P | Q | V | S | N |
| Y10^a^ | 256 (512) | **256 (256)** |  | I | N | S | M | I | L | I | A | P | Q | V | S | N |
| CL11^b^ | 128 (64) | 256 (128) |  | I | N | S | M | I | L | I | V | H | Q | V | S | N |
| A/equine/Devon/1/2011^b^ | 128 (64) | 256 (256) |  | I | N | S | M | I | L | I | V | P | Q | V | S | N |
| A/equine/Worcestershire/2/2012^b^ | 128 (51) | 256 (256) |  | I | N | S | M | I | L | I | V | P | Q | V | S | N |

a: Fc2 viruses without the A144V substitution in antigenic site A.

b: Fc2 viruses with the A144V substitution in antigenic site A.

Homologous titers are shown in bold. VN titers are represented in parentheses.^2^

Table S2. The rectal temperatures of all horses after challenge

| Days after challenge | Y10 group | | | | |  | LP93 group | | | | |
| --- | --- | --- | --- | --- | --- | --- | --- | --- | --- | --- | --- |
|  | Horse1 | Horse2 | Horse3 | Horse4 | Horse5 |  | Horse6 | Horse7 | Horse8 | Horse9 | Horse10 |
| 0 | 38.1 | 37.7 | 38.0 | 38.0 | 38.0 |  | 37.8 | 38.1 | 38.1 | 37.9 | 37.5 |
| 1 | 37.8 | 37.9 | 37.9 | 38.0 | 37.7 |  | 37.8 | 38.2 | **38.5** | 37.8 | 37.9 |
| 2 | 37.9 | 37.8 | 38.0 | 38.1 | **39.1** |  | 38.4 | **39.0** | **38.8** | **38.5** | 38.0 |
| 3 | 37.9 | 37.7 | 37.8 | 37.8 | 38.0 |  | 38.2 | **38.5** | 38.4 | 37.9 | 38.0 |
| 4 | 38.0 | 37.6 | 37.9 | 38.1 | **38.5** |  | 37.8 | **38.5** | **38.5** | 38.1 | 37.9 |
| 5 | 37.8 | 37.8 | 37.8 | 38.2 | 37.8 |  | 37.8 | 38.4 | 38.3 | **38.6** | **38.5** |
| 6 | 38.0 | 37.6 | 38.0 | 37.9 | 37.7 |  | 37.8 | 38.0 | 38.2 | **38.5** | **39.0** |
| 7 | 37.9 | 37.6 | 37.8 | 38.1 | 37.8 |  | 37.7 | 38.2 | **38.6** | 38.0 | **38.5** |
| 8 | 38.0 | 37.6 | 38.1 | 38.1 | 37.6 |  | 37.8 | 38.2 | 38.4 | 38.0 | 38.4 |
| 9 | 38.2 | 37.7 | 38.1 | 38.1 | 37.5 |  | 37.8 | 38.0 | **38.5** | 38.4 | 38.1 |
| 10 | 38.2 | 37.8 | 37.8 | 38.2 | 37.6 |  | 37.7 | 38.1 | 38.4 | 38.2 | 38.1 |
| 11 | 37.8 | 37.8 | 38.1 | 37.9 | 37.6 |  | 37.7 | 38.4 | 38.3 | 37.9 | 38.1 |
| 12 | 38.3 | 37.5 | 37.8 | 38.0 | 37.6 |  | 37.9 | 38.1 | 38.4 | 37.8 | **38.5** |
| 13 | 38.2 | 37.5 | 38.0 | 38.3 | 37.9 |  | 37.9 | 38.0 | 38.0 | 38.3 | 38.2 |
| 14 | 38.0 | 37.6 | 38.0 | 38.0 | 37.8 |  | 37.9 | 38.0 | 37.8 | 38.0 | 37.9 |

Pyrexia was defined as a rectal temperature of ≥38.5℃.

Table S3. Symptoms displayed by individual horses after challenge

| Days after challenge | Y10 group | | | | |  | LP93 group | | | | |
| --- | --- | --- | --- | --- | --- | --- | --- | --- | --- | --- | --- |
|  | Horse1 | Horse2 | Horse3 | Horse4 | Horse5 |  | Horse6 | Horse7 | Horse8 | Horse9 | Horse10 |
| 0 | - | - | - | S^a^ | - |  | - | - | S | - | S |
| 1 | - | - | - | - | - |  | S | - | - | - | S |
| 2 | - | - | - | - | - |  | - | - | S | S | M |
| 3 | - | - | - | - | - |  | - | S | S | S | M |
| 4 | - | - | - | - | M^b^ |  | - | S, C | M | M, C | M |
| 5 | - | - | - | - | M, C^c^ |  | - | S | M | S | M, C |
| 6 | - | - | - | - | S |  | S | - | S | S | S |
| 7 | - | - | - | - | - |  | - | C | M | - | S |
| 8 | - | - | - | M | M |  | S | S | M | M | M |
| 9 | - | - | - | - | M |  | - | - | S | S | M |
| 10 | - | - | - | M | - |  | - | S | M | - | M |
| 11 | - | - | C | C | M |  | - | - | S | - | S |
| 12 | - | - | - | S | M |  | - | - | S | S | M, C^c^ |
| 13 | - | - | - | - | - |  | - | - | - | S | M |
| 14 | - | - | - | - | - |  | - | - | S | - | M |

a: serous nasal discharge. b: mucous nasal discharge. c: coughing (2-5 times for ten min)

Mean durations (days) of prevalence of clinical signs between groups were significantly different (P=0.045, unpaired Student’s *t*-test).
